# Seed tissue and nutrient partitioning, a case for the nucellus

**DOI:** 10.1007/s00497-018-0338-1

**Published:** 2018-06-05

**Authors:** Jing Lu, Enrico Magnani

**Affiliations:** 1 Institut Jean-Pierre Bourgin, INRA, AgroParisTech, CNRS, University of Paris-Saclay, Route de St-Cyr (RD10), 78026 Versailles Cedex, France; 2 Ecole Doctorale 567 Sciences du Végétal, University Paris-Sud, University of Paris-Saclay, Bat 360, 91405 Orsay Cedex, France

**Keywords:** Ovule, Seed, Nucellus, Perisperm, Endosperm, Partitioning

## Abstract

Flowering plants display a large spectrum of seed architectures. The volume ratio of maternal versus zygotic seed tissues changes considerably among species and underlies different nutrient-storing strategies. Such diversity arose through the evolution of cell elimination programs that regulate the relative growth of one tissue over another to become the major storage compartment. The elimination of the nucellus maternal tissue is regulated by developmental programs that marked the origin of angiosperms and outlined the most ancient seed architectures. This review focuses on such a defining mechanism for seed evolution and discusses the role of nucellus development in seed tissues and nutrient partitioning at the light of novel discoveries on its molecular regulation.

## Introduction

**Fig. 1:**
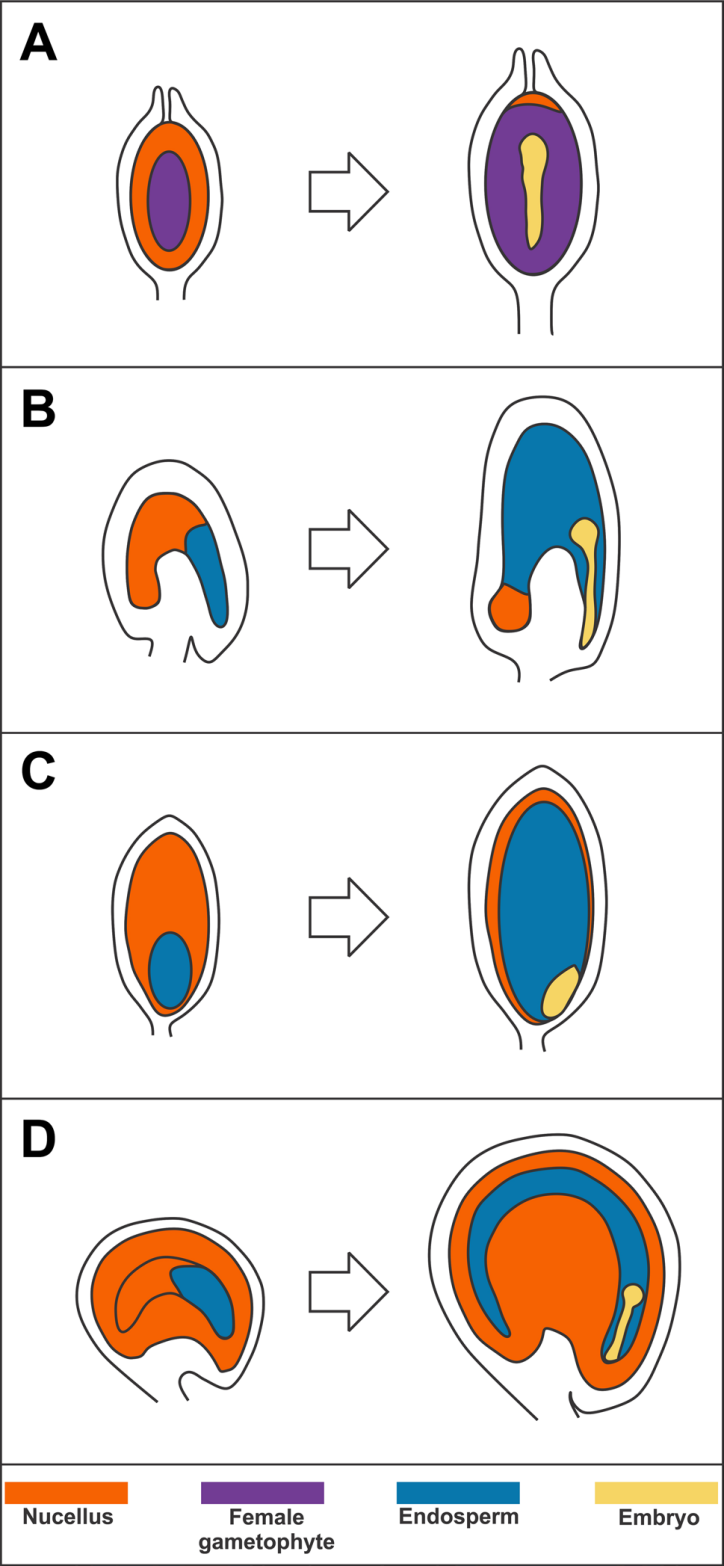
Seed architectures. Diagrammatic representation in longitudinal sections of pine (gymnosperm) (**a**), Arabidopsis (angiosperm, endospermic) (**b**), rice (angiosperm, endospermic) (**c**), and quinoa (angiosperm, perispermic) (**d**) seeds right after fertilization and at an early embryogenesis stage. The figure is not in scale. Female gametophyte, nucellus, endosperm, and embryo are highlighted in violet, orange, blue, and yellow, respectively

Tissue partitioning is the driving force that shapes the development of different seed structures. The relative contribution of each tissue to the final seed mass varies considerably among species and underlies different nutrient-storing strategies. Tissue partitioning is achieved through cell elimination programs that regulate the degeneration of one tissue in favor of another (Ingram 2017). The nucellus, the most distal maternal tissue of the ovule primordium (the seed precursor) responsible for the formation of the female gametophyte, plays a key role in defining the seed structure together with the fertilization product/s. In gymnosperms, most of the nucellus is eliminated and replaced by the female gametophyte, the main storage tissue, which will be in turn absorbed by the developing embryo, the only fertilization product (Fig. 1) (Linkies et al. 2010). Angiosperm seeds have been classified into three major architectures according to the relative volumes of the fertilization products, embryo and endosperm, and the nucellus (Fig. 1). In mature endospermic seeds (e.g., cereals), the endosperm surrounds the embryo and plays an important role in nutrient storing (Sreenivasulu and Wobus 2013). By contrast, the endosperm of non-endospermic seeds (e.g., most legumes) is completely consumed by the embryo, which becomes the primary storage tissue (Weber et al. 2005). Finally, perispermic seeds (e.g., pseudocereals such as amaranth and quinoa) develop a large perisperm, a tissue originating from the nucellus, along with a minute endosperm (Burrieza et al. 2014). The ancestral condition of angiosperm seeds is still debated between endospermic and perispermic as basal angiosperms display either a large nucellus or endosperm as primary seed storage compartment (Friedman and Bachelier 2013). Plants shifted several times between the endospermic and perispermic seed condition highlighting the antagonistic development of endosperm and nucellus as a defining mechanism for seed evolution.

Recent discoveries on the molecular regulation of nucellus elimination have given an insight into the process of seed tissues partitioning. Here, we discuss them in the context of angiosperm seed natural diversity. Finally, we review nutrient transport and accumulation in the nucellus across different seed architectures to present seed tissue and nutrient partitioning as two coherent and inextricably linked aspects of seed development.

## Natural diversity in nucellus morphology

Angiosperm ovules have been classified according to their nucellus position and thickness (Endress 2011). A first general distinction is made between ovules that bear nucellus hypodermal cells above the megaspore mother cell (MMC) (crassinucellar) and those that display only distal epidermal nucellus cells (tenuinucellar). Crassinucellar ovules are considered ancestral to tenuinucellar, as they are present in basal angiosperms, magnoliids, most monocots, and basal and part of the core eudicots. They are further classified into (1) truly crassinucellar, if they carry two or more distal hypodermal nucellus cell layers, (2) weakly crassinucellar when they display only one hypodermal cell layer, or (3) pseudo-crassinucellar if the distal nucellus epidermal cell layer divides periclinally to form additional cell layers, in the absence of hypodermal cells. The tenuinucellar condition, observed in several monocots and part of the core eudicots, includes (4) incompletely tenuinucellar ovules, which display hypodermal nucellus cells proximal and/or lateral to the MMC, (5) truly tenuinucellar ovules, without any hypodermal nucellus cell, and (6) reduced tenuinucellar ovules, when the proximal region of the MMC is not fully enclosed by the nucellus. Further terminology has been created to describe specific nucellus regions. In pseudo-crassinucellar ovules, the dermal layers of the nucellus apex (at the micropylar region) undergoing periclinal cell divisions are called “nucellar cap.” In extreme cases, the nucellus apex divides massively to form a “nucellar beak” that can extend outside the seed coat and define the micropyle. Nucellus epidermal cells can also elongate radially around the female gametophyte to form a so-called nucellar pad (Johri et al. 2013). A persistent nucellus base, at the chalazal side, is instead referred to as “podium” or “postament” if only its axial part persists (Johri et al. 2013). Overall, this classification highlights the great natural diversity in ovule nucellus size, which sets the premises for tissue partitioning programs later on in development.

Nucellus architecture changes during ovule and seed development. The female gametophyte grows at the expense of the nucellus which is partially eliminated, a process that is still almost completely unexplored (Johri et al. 2013). After fertilization, the nucellus of endospermic and non-endospermic seeds partially degenerates to make space to endosperm and embryo growth. By contrast, perispermic seeds display a large central nucellus (perisperm) that grows to become the main storage tissue along a minute endosperm. Variations have been observed in between these extreme seed architectures. A retard in the elimination of the nucellus is a hallmark of coffee grains. In coffee, the nucellus grows to define seed size and is then replaced by the endosperm (Alves et al. 2016; Mayne 1937). Similarly, the nucellus of *Austrobaileya scandens* seeds drives early seed growth and is then eliminated by the endosperm, whose further development determines final seed size (Losada et al. 2017). By contrast, nucellus and endosperm coexist and display a similar volume in *Acorus calamus* seeds (Floyd and Friedman 2000). Furthermore, the structure of *Malpighiaceae* seeds appears perispermic during early seed development but the nucellus is fully eliminated by the embryo at later stages (Souto and Oliveira 2014). Finally, *Podostemaceae* ovules do not undergo central cell fertilization and lack an endosperm. In these species, the nucellus cell walls proximal to the female gametophyte break down to produce a multinucleate cytoplasmic structure termed “nucellar plasmodium” (Arekal and Nagendran 1975, 1977).

A mechanical role for the nucellus has also been hypothesized. The anticlinal cell walls of the rice nucellus epidermis, surrounding the endosperm, are uniquely thickened with cellulosic material and have been speculated to provide mechanical support (Krishnan and Dayanandan 2003). Similarly, the chalazal or micropylar nucellus cells can differentiate into the so-called hypostase and epistase, respectively. The cell walls of these nucellar structures thicken and accumulate cutin, suberin, lignin, or callose. Hypostase and epistase have not been assigned a clear function yet but are thought to play a mechanical role or work as apoplastic barriers (Johri et al. 2013).

## Tissue partitioning

### Nucellus elimination in Arabidopsis

In Arabidopsis seeds, nucellus elimination begins 2 days after flowering (DAF) and progresses in a distal–proximal fashion to achieve the loss of 50% of its cells by 8 DAF. A few layers of proximal nucellus cells persist and expand with the rest of the ovules to form a gate between chalaza and endosperm till embryo maturity (Xu et al. 2016).

**Fig. 2:**
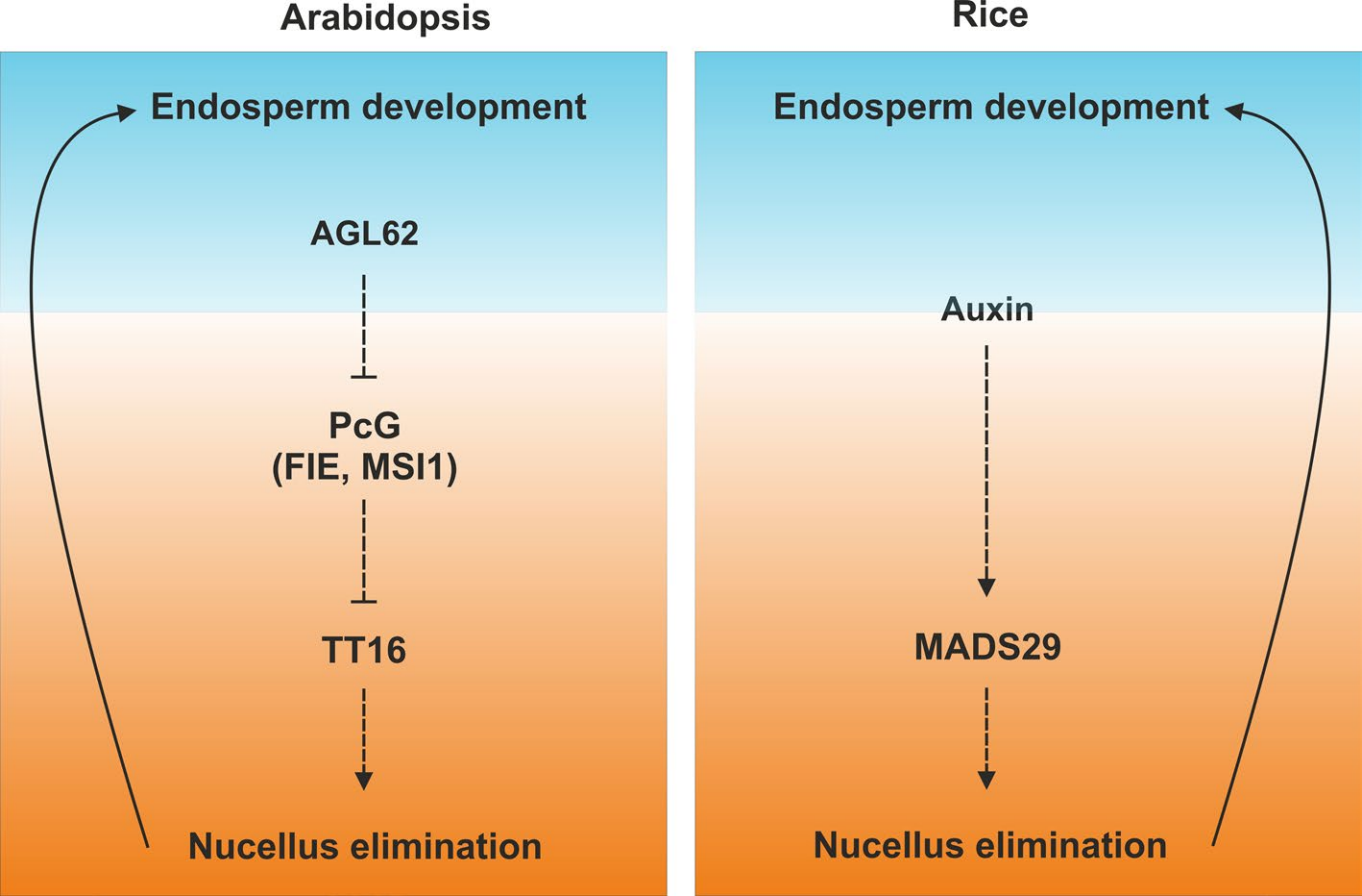
Signaling pathways underlying nucellus and endosperm antagonistic development. Arrows indicate functional relationships

Elimination of the nucellus, as well as seed coat growth, is triggered by the endosperm (Fig. 2) (Roszak and Kohler 2011; Xu et al. 2016). Single fertilization of the central cell is necessary and sufficient to initiate nucellus degeneration. The MADS box transcription factor AGAMOUS LIKE 62 (AGL62) is specifically expressed in the endosperm and essential for nucellus–endosperm communication. *agl62* mutant seeds display precocious endosperm cellularization and fail to undergo nucellus degeneration and seed coat differentiation. Figueiredo and co-workers have recently proposed that AGL62 regulates auxin efflux, considered the fertilization signal that coordinates the development of endosperm and maternal tissues (Figueiredo et al. 2016). Nevertheless, this model has been tested solely on seed coat growth and not on nucellus degeneration. Two alternative scenarios have been proposed to explain nucellus elimination: The endosperm might generate mechanical signals while growing against the nucellus or act as strong nutrient sink, thus triggering death of neighboring tissues by nutrient deprivation (Ingram 2017). It has been argued that the latter two models are less favorable to explain endosperm-maternal tissue developmental coordination as *titan 2* mutant seeds, which undergo early endosperm arrest comparable to *agl62* (Liu and Meinke 1998), show signs of seed coat growth (Roszak and Kohler 2011) and nucellus degeneration (personal observations).

Regardless of the nature of the signaling mechanism, it has been shown that endosperm growth relieves the repressive action mediated by Fertilization-Independent Seed (FIS) Polycomb Group (PcG) proteins on nucellus degeneration (Xu et al. 2016). Compared to other FIS genes that are solely expressed in the ovule central cell, *Fertilization-Independent Endosperm* (*FIE*) and *Multicopy Suppressor of IRA1* (*MSI1*) are also expressed in the nucellus and seed coat (Kohler et al. 2003; Xu et al. 2016). Both *fie/*+ and *msi1/*+ mutants display high penetrance of autonomous seed coat growth (Roszak and Kohler 2011) and nucellus degeneration (Xu et al. 2016) in the absence of fertilization. Downstream of PcG proteins, TRANSPARENT TESTA 16 (TT16) and GORDITA (GOA) MADS BOX transcription factors promote nucellus elimination and inhibit cell division (Xu et al. 2016). TT16 regulates nucellus cell elimination in part by repressing the expression of *HVA22d*, which inhibits gibberellin-mediated programmed cell death (PCD) and autophagy. Furthermore, a papain-type KDEL-tailed cysteine endopeptidase (CysEP), involved in PCD of vegetative tissues, has been shown to be expressed in the distal nucellus undergoing degeneration (Zhou et al. 2016). Nevertheless, nucellus elimination has not been entirely assigned to any known cell death program. As in vacuolar PCD (van Doorn et al. 2011), nucellus cells undergo autophagy. By contrast, the nucellus displays protoplast shrinkage and largely unprocessed cell corpses, which are hallmarks of necrosis (van Doorn et al. 2011). Another example of PCD that combines signs of vacuolar and necrotic cell death is induced by the successful recognition of pathogens during hypersensitive response (HR) (van Doorn et al. 2011). Nevertheless, PCD associated with HR does not exhibit degradation of the cell wall as in the nucellus. Furthermore, mutations in the *METACASPASE1* and *LESION SIMULATING DISEASE1* genes, which encode components of the HR-PCD machinery (Coll et al. 2010), do not affect nucellus development (Xu et al. 2016).

As endosperm growth is necessary to initiate nucellus elimination, the persistence of the nucellus in *tt16* mutant seeds negatively affects endosperm development revealing an antagonistic development of endosperm and nucellus (Xu et al. 2016). This antagonism is reflected in the evolution of the two most ancient seed structures, perispermic and endospermic, which rely on nucellus or endosperm as major storage tissue, respectively.

### Nucellus elimination in cereals

In cereals, the nucellus accounts for most of the grain volume at anthesis and it is eliminated after fertilization in a centripetal fashion. At grain filling, only the outermost nucellus cell layer (nucellus epidermis) and a few nucellus cell layers overlaying the ovule vascular trace at the chalazal side are retained and undergo PCD more or less rapidly according to the species. The chalazal nucellus of maize grains appears as compact layers of dead cells with limited plasmodesmata connections (Felker and Shannon 1980; Kladnik et al. 2004). In sorghum, the chalazal nucellus consists of a few large cell layers which are reduced to one during development and whose symplastic connection with the chalaza is interrupted (Dwivedi et al. 2014; Maness and McBee 1986; Wang et al. 2012). Rice, *Brachypodium*, barley, and wheat grains develop instead the so-called nucellar projection, a tissue dedicated to nutrient transport simplastically connected to the placenta (Krishnan and Dayanandan 2003; Opanowicz et al. 2011; Oparka and Gates 1981; Radchuk et al. 2009, 2011; Wang et al. 1994a, b, 1995; Zheng and Wang 2011). The nucellar projection of barley and wheat grains is more developed and has been divided into different regions based on cell morphology: (starting from the integument inward) (1) actively dividing cells, (2) elongating cells, (3) transfer cells with wall ingrowth, and (4) cell debris (Radchuk et al. 2006; Thiel et al. 2008; Wang et al. 1994a; Zheng and Wang 2011). By contrast, the nucellus epidermis of rice and *Brachypodium* appears larger and more persistent, compared to other cereals (Ellis and Chaffey 1987; Opanowicz et al. 2011; Oparka and Gates 1981). Finally, the chalazal nucellus physically touches the endosperm in maze, *Brachypodium,* and rice, while it is separated by a cavity filled with nucellar lysate (referred to as “endosperm or nucellar cavity” or “placental sac”) in wheat, barley, and sorghum.

The Arabidopsis signaling pathway underlying nucellus development is partially conserved in rice grains (Fig. 2). The rice *TT16* orthologous gene, *MADS29*, is expressed in the nucellus and nucellar projection and promotes cell elimination (Nayar et al. 2013; Yang et al. 2012; Yin and Xue 2012). Compared to Arabidopsis, *MADS29* is also expressed in the embryo and the protein has been detected in nucellus epidermis, embryo, and endosperm but not in the nucellar projection (Nayar et al. 2013). MADS29 directly activates the expression of nucleotide-binding site–leucine-rich repeat proteins and Cys proteases (Yin and Xue 2012). In line with the Arabidopsis endosperm-maternal tissue signaling model (Figueiredo et al. 2016), *MADS29* expression is induced by auxin and regulates auxin–cytokinin homeostasis (Nayar et al. 2013; Yin and Xue 2012). Furthermore, antagonistic development of nucellus and endosperm has been observed also in rice as suppression of *MADS29* expression impairs starch accumulation and endosperm growth (Nayar et al. 2013; Yang et al. 2012; Yin and Xue 2012).

In barley grains, nucellus elimination correlates with the expression of genes encoding for Asp protease-like protein nucellin, vacuolar processing enzyme nucellain, Cys and Asp endopeptidases, subtilisin-like Ser proteinases, and JEKYLL protein, all known to play a role in PCD (Chen and Foolad 1997; Linnestad et al. 1998; Radchuk et al. 2006, 2011, 2018; Thiel et al. 2008; Tran et al. 2014). Down-regulation of *jekyll* by RNA interference affects nucellus elimination and nucellar projection differentiation and, indirectly, endosperm development and starch accumulation (Radchuk et al. 2006). Furthermore, the differentiation gradient along the barley nucellar projection is also regulated by a gibberellin-to-abscisic acid balance, with gibberellin promoting differentiation (Weier et al. 2014).

Morphological analyses of nucellus parenchymal cells in wheat revealed fragmentation of the cytoplasm, vacuolization, disruption of the nuclear envelope and plasma membrane, and mitochondrion structural alterations (Dominguez et al. 2001). Nevertheless, the authors of this study might have erroneously located nucellus cells as what it is indicated as nucellus parenchymal cell in Fig. 2b appears to be integument cells. In line with this interpretation, the cells analyzed do not undergo degeneration of the cell wall. Nucellus epidermis and nucellar projection of wheat grains have been shown to express genes encoding for carboxypeptidase III, thiol protease, nucellain, and nucellin, some of which are also implicated in aleurone death during germination (Domınguez and Cejudo 1998; Drea et al. 2005). A parallel has been drawn between wheat and *Brachypodium* nucellus, which also expresses nucellain during its elimination (Opanowicz et al. 2011).

Finally, the study of the maize invertase *Miniature 1* (*Mn1*) gene revealed a mechanical interaction between nucellus and endosperm. Maize grains mutated for the *Mn1* gene show a gap between the nucellus cells, which are rapidly emptied of their nuclear and cytoplasmic material, and the endosperm. Such a gap is not due to cell death but to an underdeveloped endosperm that results in over-expanded nucellus cells, thus suggesting that the endosperm exercises a mechanical force on the nucellus (Kladnik et al. 2004).

Overall, these data indicate that a protease-dependent cell death machinery is shared by cereals to achieve nucellus degeneration. These same types of proteases, even though not necessarily the same genes, appear to drive endosperm cell death. On the other hand, more data are necessary to highlight variations in the nucellus elimination pathways responsible for the slightly different nucellus fates observed in different cereals.

### Nucellus elimination in other angiosperms

Similar to Arabidopsis and cereals, a number of other angiosperm seeds have been shown to undergo early nucellus elimination in a progressive fashion starting from the nucellus–endosperm border toward the chalazal region. Proteomics and genetic analyses revealed the presence of Cys endopeptidases and other peptidases associated with PCD in the nucellus of castor bean seeds (Greenwood et al. 2005; Nogueira et al. 2012). Cys endopeptidases are accumulated in ricinosomes, organelles derived from the endoplasmic reticulum that collapse upon nucellus degeneration releasing their content in the cytoplasm and contributing to the digestion of proteinaceous debris (Greenwood et al. 2005). In *Sechium edule*, nucellus elimination correlates with the induction of caspase-like proteases and high levels of hydrogen peroxide, nitric oxide, and ethylene, which has been proposed as the signaling molecule between endosperm and nucellus (Lombardi et al. 2007, 2010, 2012). High level of indole acetic acid has also been detected in endosperm and nucellus of *Sechium edule* seeds but its role in nucellus development is still unclear (Lombardi et al. 2012). By contrast, the nucellus of peach seeds displays a pick of abscisic acid after anthesis, thus suggesting that different hormones might play a role in nucellus degeneration in different species (Piaggesi et al. 1991).

### Nucellus retention

Perispermic seeds such as quinoa, amaranth, *Peperomia*, spinach, and *Nymphaeales* display a large nucellus, which defines seed size and becomes the major storage tissue, along a minute endosperm. The process has been well studied in quinoa. At anthesis, the nucellus reaches its final number of cells as its mitotic activity arrests. After fertilization, a relatively small endosperm grows at the expense of part of the nucellus and leads the way to embryo development which in turn consumes most of the endosperm and part of the nucellus. The central nucellus, termed perisperm, is not eliminated and undergoes cell expansion, endoreduplication, reserve accumulation, and PCD. Nucellus cell death involves nuclease and proteolytic activity but not cell wall degeneration, a process comparable to endosperm cell death in endospermic seeds (Burrieza et al. 2014; Lopez-Fernandez and Maldonado 2013).

## Nutrient partitioning

Tissue and nutrient partitioning are two inextricably linked processes. Such a diverse panorama of seed structures correlates therefore with an equally broad spectrum of nutrient-storing strategies. What all angiosperm seeds have in common is the allocation of resources from the placental maternal tissue, through the chalaza, to the storage tissues following a source-sink nutrient gradient (Patrick and Offler 2001). In most angiosperms, vascularization arrests at the chalaza, and nutrients follow a combination of symplastic and apoplastic pathways to reach the sink tissues. Nevertheless, there are examples of nucellar tracheids, an ancestral character also observed in extinct gymnosperms, and vascularized seed coats (Johri et al. 2013).

### Sugar transport in endospermic seeds

The role of the nucellus in nutrient transport has been mostly addressed studying cereal grain filling. In cereals, nutrients are supposed to travel simplastically from the phloem through the maternal tissues of the chalazal region to then being released into the apoplast. The endosperm, which is not simplastically connected to the maternal tissues, uploads nutrients from the apoplast and accumulates mostly starch while undergoing PCD (Thorne 1985). The nucellus lies at the interface of maternal and endosperm tissues and can play a role in nutrient transfer.

In maize grains, sucrose moves simplastically from the phloem to the chalaza and is then released into the apoplast where cell wall-bound invertases convert it into hexoses, glucose and fructose (Felker and Shannon 1980; McLaughlin and Boyer 2004; Porter et al. 1985; Shannon 1972a, b; Tang and Boyer 2013). The nucellus is not simplastically connected to the chalaza and imports glucose during the first stages of grain development while being eliminated by endosperm growth (McLaughlin and Boyer 2004; Tang and Boyer 2013). Later in development, persistent nucellus cells undergo PCD (Felker and Shannon 1980; Kladnik et al. 2004), thus suggesting that nutrients cross the nucellus apoplastically. A similar path of sugar transport probably occurs in sorghum grains as they accumulate hexoses in the placental sac and display symplastic disconnection of chalaza and nucellus (Dwivedi et al. 2014; Maness and McBee 1986; Wang et al. 2012).

By contrast, the nucellus of wheat and barley is simplastically connected to the placenta and the nucellar projection develops transfer cells, thus expanding the nutrient unloading zone and facilitating transfer (Radchuk et al. 2009, 2011; Wang et al. 1994a, b, 1995; Zheng and Wang 2011). At the beginning of barley seed development, starch accumulates mostly in the pericarp, which acts as a short-term sink, and only transiently in the nucellus. Alpha amylase 4 is expressed in degenerating nucellus tissue facilitating mobilization of starch toward the endosperm during nucellus elimination (Radchuk et al. 2006, 2009). At barley grain filling, ^13^C sucrose analyses revealed a flow of sucrose from the nucellar projection toward the endosperm (Melkus et al. 2011; Rolletschek et al. 2011). The nucellus projection of barley grains expresses a cell wall-bound invertase, indicating that hexoses are also released into the endosperm cavity (Weschke et al. 2003). Furthermore, barley nucellar projection and epidermis express members of the aquaporin family, which may play a role in nutrient efflux (Thiel et al. 2008). Interestingly, transfer cells of the nucellar projection and endosperm of barley and wheat express the same sucrose symporter (*SUT*) genes responsible for sucrose import in sink tissues (Bagnall et al. 2000; Weschke et al. 2000). The role of SUT proteins in the nucellus is not clear, and it might allow sucrose scavenging, work as sucrose passive port along concentration gradient or be an evolutionary relic of perispermic seeds. Impaired development of the barley nucellar projection leads to starch accumulation in maternal tissues at the expense of the endosperm, thus further proving the importance of this tissue in nutrient partitioning (Melkus et al. 2011; Radchuk et al. 2006; Rolletschek et al. 2011).

Rice and *Brachypodium* grains develop a smaller nucellar projection than barley and wheat, but display a thicker nucellus epidermis which has been proposed to play an active role in nutrient transport (Ellis and Chaffey 1987; Opanowicz et al. 2011; Oparka and Gates 1981). Defective starch synthesis in the endosperm has been observed in rice grains with suppressed *MADS29* expression, highlighting the active role of the rice nucellus in transferring nutrients to the endosperm (Nayar et al. 2013; Yin and Xue 2012). During nutrient transfer, the nucellus might act as a short-term sink as MADS29 has been found to promote the differentiation of proplastids in amyloplasts likely by regulating cytokinin biosynthesis (Nayar et al. 2013). Finally, SWEET sucrose exporters have been found in all rice nucellar tissues, indicating that the nucellus engages in apoplastic seed filling (Yang et al. 2018).

### Sugar transport in perispermic seeds

The role of the nucellus in perispermic seeds changes from nutrient-transport facilitator to long-term nutrient sink. The perisperm of quinoa seeds accumulates mostly starch while undergoing PCD (Lopez-Fernandez and Maldonado 2013). Starch accumulation follows an apical–basal pattern, with the chalazal side being the last to be filled. Such a pattern might be the result of sugars transport from the chalaza toward the perisperm while maximizing seed filling by following a source-sink gradient. Alternatively, nutrient transport through the seed coat might also explain such a nutrient accumulation pattern. At seed maturity, perisperm cells appear as thin walled and completely filled with starch grains, similar to cereal starchy endosperm cells (Lopez-Fernandez and Maldonado 2013; Prego et al. 1998). By contrast, the role of the few endosperm cells that persist at the micropylar region is less clear.

## Conclusive remarks

The evolution of seed storage tissues in angiosperms has been a “battle” between endosperm and nucellus development. Both tissues can store starch and become the main source of energy for embryo germination. Indeed, the nucellus of perispermic seeds parallels the endosperm of endospermic seeds at both morphological and functional levels. Nevertheless, most angiosperm seeds evolved mutually exclusive growth of nucellus and endosperm. The nucellus offers an easier system of nutrient storage simplastically connected to the placenta. By contrast, the endosperm couples nutrient storing to fertilization, thus possibly avoiding energy waste in case of unsuccessful fertilization.

Whereas we have a better understanding on seed nutrient transport and tissue elimination, the next challenge will be to address how nutrient and tissue partitioning are coordinated at the molecular level.

### Author contribution statement

JL reviewed the role of MADS box genes in nucellus development. EM wrote the rest of the paper in consultation with JL.
